# Personal values in adolescence and suicidality: a cross-sectional study based on a retrospective recall

**DOI:** 10.1186/s12888-019-2194-4

**Published:** 2019-07-09

**Authors:** Naonori Yasuma, Kazuhiro Watanabe, Asami Matsunaga, Daisuke Nishi, Norito Kawakami

**Affiliations:** 1 Department of Mental Health, Graduate School of Medicine, The University of Tokyo, 7-3-1 Hongo, Bunkyo-ku, Tokyo, 1130033 Japan; 2 Department of Community Mental Health and Law, National Institute of Mental Health, National Center of Neurology and Psychiatry, Kodaira, Japan

**Keywords:** Suicidality, Personal values, Commitment to values

## Abstract

**Background:**

This study retrospectively examined the association between personal values in adolescence and suicidality in a community-representative adult sample in Japan.

**Methods:**

Data were used from wave 1 (2010) and wave 3 (2017) of a Japanese Study on Stratification, Health, Income, and Neighborhood (J-SHINE) survey. Personal values at age 15 were retrospectively measured in 2017 in two ways: the original value priorities we made were based on the Schwartz theory of basic values; and the Japanese version of the Personal Values Questionnaire II (PVQ-II) was used to assess the degree of commitment to the values. Multivariate logistic regression analysis was used to estimate the association of personal values in adolescence with self-reported suicidal ideation, suicidal planning, and suicide attempt (both during a lifetime and in the most recent year), adjusting for socio-demographics, smoking, alcohol consumption, and economic status among 15 year olds.

**Results:**

Cherishing family and friends was significantly and negatively associated with suicidal ideation over a lifetime and in the most recent year. Commitment to values was also significantly and negatively associated with suicidal ideation in the most recent year.

**Conclusions:**

Investigating the association between personal values and suicidality could make a significant contribution to the literature by offering a new approach to understanding and preventing suicide.

## Background

Suicide is a major public health problem all over the world [1]. Globally, around 0.8 million people die of suicide, and even more people attempt to end their lives every year [2]. According to a World Health Organization survey, the average 12-month prevalence of suicidal ideation is 2.0% in high-income countries and 2.1% in low-income countries [3]. The lifetime prevalence of suicidal ideation is 9.2% and that of attempted suicide is 2.7% in the world [4]. In 2017, the number of people who committed suicide in Japan was 21,321 [5], and the lifetime prevalence of suicidal ideation, planning, and attempt was 10.9, 2.1, and 1.9%, respectively [6]. The socio-economic impact of suicide is huge [7] and the psychological burden of suicide on family and friends is serious [8].

Suicide is associated with various individual psychological and behavioral factors. For example, past suicide attempts [9], past history of mental illness, such as depression [10] or personality disorder [11], and alcohol or drug abuse were associated with suicide [12]. Hopelessness [13], isolation [14], lack of social support [15], unemployment, and economic loss have also been related to suicide [16]. In addition, spending one’s childhood in a dysfunctional family in which divorce or the separation of parents has taken place, or being physically, sexually, or psychologically abused have been associated with a higher risk of suicide [17]. It has been found that adversity during childhood leads to various adaptation problems during adolescence and less coping skills, which increase the risk of suicide [18, 19].

Personal values are defined as broad goals of varying in importance that underlie and guide attitudes and behavior [20, 21]. Adolescence is a period of substantial psychological and emotional development, wherein the brain’s reward system is remodeled [22]. Personal values developed during adolescence may affect long-term cognitions, behaviors, and finally health and well-being. Personal values have usually been studied in two components: the content of values [23] and commitment to values [24]. Previous studies have indicated that the content of values is associated with various indicators of health and well-being [25, 26]; and commitment to values is also associated with well-being [27, 28]. Personal values developed in adolescence could also be an important factor for suicidality in a lifetime. For instance, having some areas of value priorities may prevent people developing suicidal thoughts and behaviors, because such value areas may be associated with better social adjustment in adulthood or less isolation from society [29]. A stronger commitment to values may facilitate one’s ability to cope with suicidal thoughts [23]. Accordingly, personal values in adolescence could be associated with suicidality. The promotion of mental health in adolescence has attracted much attention because this period is believed to determine the psychological and behavioral factors of adolescents in later life [22]. Therefore, understanding the association between personal values in adolescence and suicidality could be useful in predicting or preventing suicide.

An important objective of this study was to establish whether there is any association between personal values in adolescence and adult suicidality (suicidal ideation, suicidal planning and suicide attempt). This assessment was conducted retrospectively by analyzing existing data collected from a large community sample in Japan.

## Methods

### Study design, setting and participants

**Fig. 1:**
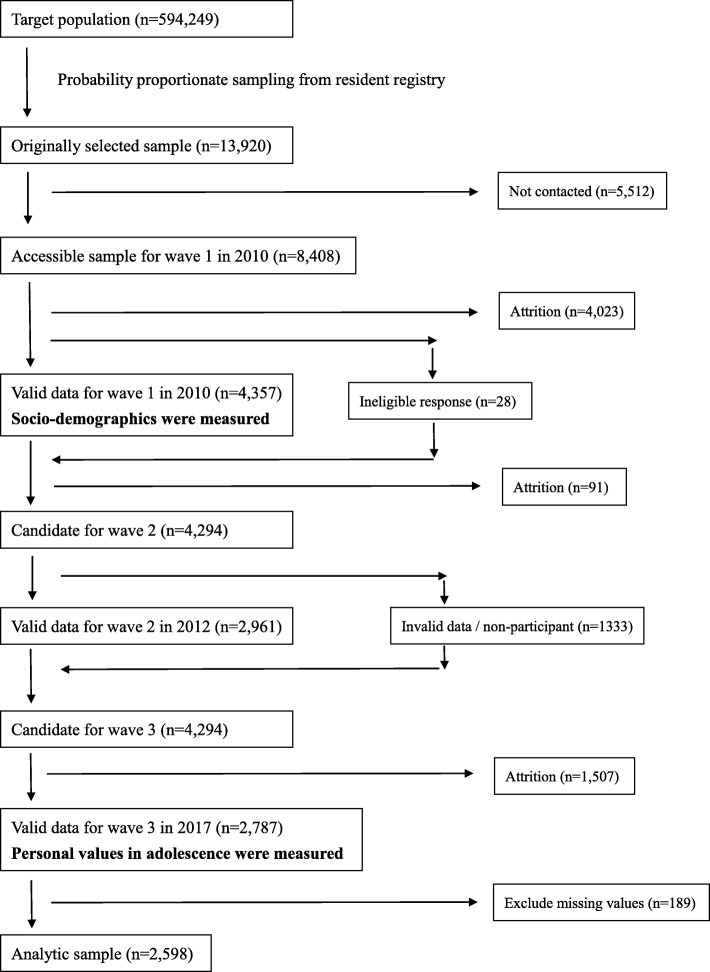
Flowchart of participant recruitment in J-SHINE

This study is cross-sectional and based on a retrospective recall using wave 1 and wave 3 data from a Japanese Study on Stratification, Health, Income, and Neighborhood (J-SHINE) survey [30]. The J-SHINE survey was a panel study conducted to clarify the complex associations between social factors and health. The sample was randomly selected from adult residents aged 20 to 50 years from four municipalities (two in Tokyo; two in neighboring prefectures) using systematic sampling methods from a residents’ register. There were no inclusion and exclusion criteria except for age. Invitation letters were sent to the participants, and trained surveyors visited their houses. The participants were asked to provide written informed consent and answered the self-administered questionnaire with a computer-aided personal instrument (CAPI). Three investigations have been conducted to date (Fig. 1). The wave 1 survey was conducted in 2010. Of the 13,920 people originally selected, 4357 responded to the questionnaire (% valid response: 31.3%). The wave 2 survey was carried out in 2012 and those who responded to wave 1 were recruited. Among the 4294 candidates eligible to participate in the study, 2961 responded (% valid response: 69.0%). The wave 3 survey was conducted in 2017, and those who responded to both the wave 1 and wave 2 survey were selected. Two thousand seven hundred eighty-seven people answered the questionnaire (% valid response: 64.9%). Suicidality and socio-demographics such as age, gender, marital status, education, employment, household income, smoking, drinking alcohol, and economic status at age 15 were measured in wave 1, and personal values in adolescence were measured in wave 3. The study protocol was approved by the Research Ethics Committee of the Graduate School of Medicine and the Faculty of Medicine, The University of Tokyo, Japan [No.630–73,361]. This article complies with the Strengthening the Reporting of Observational Studies in Epidemiology (STROBE) guidelines [31].

## Measures

### Personal values in adolescence

Personal values in adolescence were measured by using value priorities and degrees of commitment to values. To measure value priorities, we developed 11 items based on the 57-item Portrait Values Questionnaire (PVQ-57; [29]). Eleven value orientations were as follows: avoiding causing trouble, positive evaluation, belief, financial success, improving society, interest, social influence, enduring active challenging, cherishing family and friends, graduating from school, and stable lifestyle. These items were rated on a seven-point Likert scale (1 = Not at all, 7 = Very important) following the question, “When you were 15-16 years old, how important did you think the following values were in your life?” Commitment to values was measured by the Japanese version of the Personal Values Questionnaire II (PVQ-II) [32]. PVQ-II consists of eight items (e.g., How committed are you to living this value?); the items were rated on a five-point Likert scale. Internal consistency and concurrent and structural validity had already been confirmed. In this study, we revised the items to the past tense and instructed the participants to answer the items they considered the most important when they were 15–16 years old. The total scores on the PVQ-II were used for analysis; higher scores indicate more commitment to important values.

### Suicidality

Questions relating to suicidality were derived from WHO World Mental Health Surveys [33]. The respondents were asked three questions relating to lifetime suicidality, i.e., suicidal ideation, suicidal planning, and suicide attempt, with a dichotomous response option (yes/no) for each question: For suicidal ideation, “Have you ever seriously thought about suicide?” For suicidal planning, “Have you ever planned suicide so far?” For attempted suicide, “Have you tried suicide before?” If a respondent endorsed each of these lifetime questions, then he/she was asked if he/she had been through that experience in the most recent year.

### Socio-demographics

The questionnaire elicited socio-demographic information from the participants by asking questions related to age, gender, marital status, education, employment, household income, smoking, drinking alcohol, and economic status. Education was divided into four groups: junior high school graduates, high school graduates, some college, university graduates, or higher. Employment was divided into three groups, working, on leave, job seeking, housewife, or students. Based on the sum of annual household income, the respondents were divided into five groups: less than 2.5 million yen, from 2.5 million yen to less than 5 million yen, from 5 million yen to less than 7.5 million yen, 7.5 million yen or over, and not known. As for smoking, ex-smoker was included in smoker. Drinking alcohol was divided into two items: whether the participants were habitual drinkers (drinking more than three times per week) or not. The economic status of the 15 year olds was classified as poor, moderate, or good.

#### Sample size calculation

Sample size calculation was performed by using exponential possible correlations (ρ) between personal values in adolescence and outcomes based on previous studies [22, 26, 27, 34, 35]; the estimated minimum correlation was set to 0.1 and the odds ratio was calculated at 1.1. We set the H0 (the lifetime prevalence of suicidal ideation) at 0.2 [36] and R^2^ 0.1. Using G*Power version 3.1.9.2. [37], the required sample size was estimated to be 2458, for an α error probability of 0.05 and a power (1 - β) of 0.80. If we collected data from 2598 participants and analyzed the data, the post hoc statistical power of the analysis (1 - β) would be 0.82.

#### Analysis

Multivariate logistic regression analysis was used to estimate the association between personal values in adolescence and suicidal ideation (over a lifetime and in the most recent year), suicidal planning (over a lifetime and in the most recent year), and attempted suicide (over a lifetime and in the most recent year). Model 1 was adjusted for socio-demographics; Model 2 was additionally adjusted for smoking and drinking alcohol; Model 3 was further adjusted for economic status among 15 year olds. A *p-*value of less than 0.05 was made statistically significant. SPSS (windows version 25) was used for statistical analysis. In these analyses, we did not impute missing responses on the variables.

## Results

### Demographic and psychosocial characteristics and prevalence of suicidality

Among the total of 2787 survey respondents, 2669 completed the value priorities and PVQ-II. Some of the respondents had missing values on the demographic variables smoking, drinking alcohol, and economic status at age 15 (*n* = 71), and were excluded from the study. Two thousand five hundred ninety-eight respondents who didn’t have missing values were used for analysis. According to Table 1, the mean scores of 11 value priorities and PVQ-II were shown. In addition, the prevalence of suicidal ideation over a lifetime and in the most recent year were 17.6 and 4.8%; suicidal planning over a lifetime and in the most recent year were 5.5 and 1.5%; attempted suicide over a lifetime and in the most recent year were 3.8 and 0.7%. The respondents who completed the wave 1 and 3 surveys (*n* = 2787) were significantly more likely to be older, female, married, less educated than university graduates, with more household income, non-smokers, and who had less suicide attempts in their lifetime compared to those who completed the wave 1 survey, but did not do the wave 3 survey (*n* = 1570) (Table 5).

**Table 1:** Demographics and psychosocial characteristics of the participants (*N* = 2598)

	N (%)	Mean (SD)
Age (mean)		38.10 (7.03)
Sex (men)	1150 (44.3)	
Married (yes)	1956 (75.3)	
Education		
Junior high school	77 (3.0)	
High school	512 (19.7)	
Some college	908 (34.9)	
University or higher	1101 (42.4)	
Employment		
Working	2014 (77.5)	
On leave	60 (2.3)	
Job seeking or House wife or Students	559 (20.2)	
Household Income (per year)		
≦2.5 million yen	176 (6.8)	
≦5 million yen	411 (15.8)	
≦7.5 million yen	611 (23.5)	
>7.5 million yen	825 (31.8)	
Not known	575 (22.1)	
Current or ever smoking (yes)	1178 (45.3)	
Drinking alcohol		
More than 3 times per week	877 (33.8)	
Less than 3 times per week	1721 (66.2)	
Living in 15 years old (Poor)	489 (18.8)	
Suicidality		
Suicidal Ideation over a life time (yes)	457 (17.6)	
Suicidal Ideation in the most recent year (yes)	125 (4.8)	
Suicidal Planning over a life time (yes)	144 (5.5)	
Suicidal Planning in the most recent year (yes)	40 (1.5)	
Suicide Attempt over a life time (yes)	99 (3.8)	
Suicide Attempt in the most recent year (yes)	17 (0.7)	
Value priories 11 values orientations		
Avoiding causing trouble		5.58 (1.36)
Positive evaluation		4.90 (1.39)
Belief		4.84 (1.40)
Financial success		4.22 (1.53)
Improving society		3.77 (1.43)
Pursuing one's interest		5.13 (1.40)
Social influence		3.30 (1.41)
Enduring active challenging		4.51 (1.43)
Cherishing family and friends		5.54 (1.25)
Graduating from school		4.26 (1.67)
Stable lifestyle		4.86 (1.43)
Commitment to Values		26.34 (4.76)

### Personal values in adolescence and suicidal ideation (over a lifetime and in the most recent year), suicidal planning (over a lifetime and in the most recent year), and suicide attempt (over a lifetime and in the most recent year)

According to Table 2, cherishing family and friends was significantly and negatively associated with suicidal ideation over a lifetime and in the most recent year after adjusting socio-demographics variables (Model 1), smoking and drinking alcohol (Model 2), and economic status among 15 year olds (Model 3). As for Table 3, cherishing family and friends was also negatively and significantly associated with suicidal planning over a lifetime after adjusting Model 1, Model 2, and Model 3. However, it was not significantly associated with suicidal planning in the most recent year. In Table 4, there were not any significant relationships between personal values in adolescence and suicide attempt over a lifetime and in the most recent year.

**Table 2:** Personal values in adolescence and suicidal ideation: multivariate logistic regression analysis (N = 2598)

	Suicidal Ideation (over a life time)									Suicidal Ideation (in the most recent year)								
	Model 1			Model 2			Model 3			Model 1			Model 2			Model 3		
	OR§	95%CI	*p*	OR§	95%CI	*p*	OR§	95%CI	*p*	OR§	95%CI	*p*	OR§	95%CI	*p*	OR§	95%CI	*p*
Areas of value priority																		
Avoiding causing trouble	1.00	0.91~1.10	0.99	1.01	0.92~1.10	0.88	1.01	0.92~1.10	0.89	1.12	0.95~1.32	0.18	1.13	0.96~1.33	0.15	1.12	0.95~1.32	0.17
Positive evaluation	1.03	0.95~1.12	0.49	1.03	0.95~1.12	0.50	1.03	0.95~1.12	0.48	1.01	0.87~1.17	0.92	1.01	0.87~1.17	0.92	1.00	0.86~1.16	0.99
Belief	1.01	0.91 = 1.11	0.92	1.01	0.92~1.11	0.87	1.01	0.92~1.11	0.81	1.10	0.93~1.30	0.29	1.10	0.93~1.30	0.29	1.11	0.93~1.31	0.25
Financial success	1.08	0.99~1.18	0.09	1.07	0.98~1.17	0.12	1.06	0.97~1.15	0.24	1.20	1.02~1.40	0.03*	1.19	1.02~1.40	0.03*	1.16	0.99~1.36	0.07
Improving society	1.04	0.95~1.15	0.39	1.05	0.95~1.16	0.30	1.06	0.96~1.17	0.24	1.05	0.89~1.25	0.56	1.06	0.89~1.25	0.52	1.07	0.90~1.27	0.45
Pursuing one's interest	1.08	0.98~1.18	0.12	1.08	0.98~1.18	0.11	1.08	0.99~1.19	0.09	1.17	0.98~1.39	0.08	1.17	0.99~1.39	0.07	1.18	0.99~1.40	0.06
Social influence	0.93	0.85~1.03	0.16	0.93	0.84~1.02	0.13	0.93	0.85~1.02	0.14	0.91	0.77~1.06	0.22	0.90	0.77~1.06	0.22	0.91	0.77~1.07	0.24
Enduring active challenging	0.99	0.90~1.09	0.81	0.98	0.89~1.08	0.73	0.98	0.89~1.08	0.66	1.11	0.94~1.31	0.23	1.09	0.93~1.29	0.30	1.08	0.92~1.28	0.35
Cherishing family and friends	0.81	0.74~0.89	< 0.01**	0.81	0.74~0.89	< 0.01**	0.82	0.74~0.90	<0.01**	0.75	0.64~0.89	<0.01**	0.75	0.64~0.89	<0.01**	0.76	0.64~0.89	<0.01**
Graduating from school	0.91	0.84~0.98	0.02*	0.91	0.84~0.99	0.02*	0.92	0.85~1.00	0.05	0.88	0.76~1.01	0.06	0.88	0.76~1.01	0.07	0.90	0.78~1.03	0.12
Stable lifestyle	0.97	0.88~1.07	0.55	0.98	0.89~1.08	0.67	0.97	0.88~1.07	0.56	0.91	0.76~1.08	0.26	0.91	0.77~1.08	0.29	0.91	0.77~1.08	0.29
Commitment to values	1.00	0.98~1.03	0.75	1.00	0.98~1.03	0.74	1.00	0.98~1.03	0.90	0.95	0.91~1.00	0.04*	0.95	0.91~1.00	0.04*	0.95	0.91~0.99	0.03*

§Odds Ratio (OR) for one-point increase in the score is shown

Sociodemographic variables were adjusted for in the Model 1; Smoke and Alcohol were additionally adjusted for in the Model 2; Living in 15 years old was additionally adjusted for in the Model 3

*p < 0.05, ** *p* < 0.01

**Table 3:** Personal values in adolescence and suicidal planning: multivariate logistic regression analysis (N = 2598)

	Suicidal Planning (over a life time)									Suicidal Planning (in the most recent year)								
	Model 1			Model 2			Model 3			Model 1			Model 2			Model 3		
	OR§	95%CI	*p*	OR§	95%CI	*p*	OR§	95%CI	*p*	OR§	95%CI	*p*	OR	95%CI	*p*	OR	95%CI	*p*
Areas of value priority																		
Avoiding causing trouble	1.01	0.88~1.17	0.85	1.03	0.89~1.19	0.74	1.02	0.88~1.18	0.81	1.22	0.91~1.63	0.18	1.24	0.92~1.66	0.16	1.24	0.92~1.67	0.16
Positive evaluation	1.05	0.91~1.20	0.53	1.04	0.91~1.20	0.55	1.04	0.90~1.19	0.60	1.10	0.84~1.43	0.49	1.10	0.85~1.43	0.48	1.08	0.83~1.41	0.55
Belief	0.93	0.80~1.08	0.34	0.93	0.80~1.08	0.35	0.94	0.81~1.10	0.44	1.01	0.77~1.34	0.93	1.02	0.77~1.35	0.91	1.03	0.78~1.36	0.86
Financial success	1.18	1.02~1.36	0.03*	1.16	1.01~1.35	0.04*	1.14	0.98~1.32	0.08	1.34	1.02~1.76	0.04*	1.32	1.01~1.74	0.05	1.29	0.98~1.69	0.07
Improving society	0.98	0.84~1.15	0.81	1.00	0.85~1.17	0.96	1.01	0.86~1.18	0.91	0.90	0.67~1.21	0.48	0.92	0.68~1.24	0.57	0.93	0.70~1.26	0.65
Pursuing one's interest	1.14	0.98~1.33	0.09	1.14	0.98~1.33	0.09	1.16	0.99~1.35	0.07	1.14	0.86~1.50	0.37	1.15	0.87~1.51	0.34	1.16	0.87~1.53	0.31
Social influence	0.93	0.80~1.09	0.39	0.93	0.80~1.08	0.34	0.93	0.80~1.08	0.35	0.94	0.71~1.23	0.65	0.94	0.71~1.23	0.63	0.94	0.71~1.23	0.64
Enduring active challenging	0.97	0.84~1.13	0.73	0.97	0.83~1.13	0.67	0.96	0.82~1.12	0.59	1.11	0.84~1.47	0.45	1.08	0.82~1.43	0.60	1.07	0.81~1.42	0.63
Cherishing family and friends	0.77	0.66~0.89	<0.01**	0.76	0.66~0.88	< 0.01**	0.78	0.67~0.90	<0.01**	0.79	0.59~1.04	0.09	0.78	0.59~1.03	0.08	0.79	0.59~1.04	0.09
Graduating from school	0.89	0.79~1.01	0.08	0.90	0.79~1.02	0.10	0.92	0.81~1.04	0.19	0.77	0.61~0.98	0.03*	0.78	0.61~0.99	0.04*	0.80	0.63~1.02	0.07
Stable lifestyle	0.98	0.84~1.15	0.84	1.00	0.85~1.16	0.95	0.98	0.84~1.14	0.81	1.00	0.75~1.34	0.98	1.02	0.76~1.37	0.88	1.01	0.76~1.35	0.93
Commitment to values	1.03	0.99~1.07	0.16	1.03	0.99~1.07	0.16	1.02	0.98~1.07	0.24	0.94	0.87~1.01	0.10	0.94	0.86~1.01	0.10	0.93	0.86~1.01	0.07

§Odds Ratio (OR) for one-point increase in the score is shown

Socio-demographic variables were adjusted for in the Model 1; Smoke and Alcohol were additionally adjusted for in the Model 2; Living in 15 years old was additionally adjusted for in the Model 3

*p < 0.05, ** *p* < 0.01

**Table 4:** Personal values in adolescence and suicide attempt: multivariate logistic regression analysis (*N* = 2598)

	Suicide Attempt (over a life time)									Suicide Attempt (in the most recent year)								
	Model 1			Model 2			Model 3			Model 1			Model 2			Model 3		
	OR§	95%CI	*p*	OR§	95%CI	*p*	OR§	95%CI	*p*	OR§	95%CI	*p*	OR§	95%CI	*p*	OR§	95%CI	*p*
Areas of value priority:																		
Avoiding causing trouble	1.00	0.84~1.19	1.00	1.01	0.84~1.20	0.95	0.99	0.83~1.19	0.95	1.31	0.82~2.08	0.26	1.35	0.84~2.17	0.22	1.31	0.81~2.13	0.27
Positive evaluation	1.05	0.88~1.24	0.60	1.05	0.89~1.24	0.59	1.05	0.88~1.24	0.60	1.28	0.85~1.93	0.24	1.30	0.85~1.98	0.22	1.29	0.85~1.97	0.23
Belief	1.02	0.85~1.22	0.86	1.02	0.85~1.23	0.85	1.03	0.86~1.24	0.73	1.18	0.77~1.82	0.44	1.22	0.79~1.88	0.37	1.21	0.78~1.86	0.40
Financial success	1.19	1.00~1.42	0.05	1.18	0.99~1.40	0.06	1.14	0.96~1.35	0.14	1.37	0.90~2.07	0.15	1.35	0.89~2.05	0.16	1.33	0.88~2.02	0.18
Improving society	0.93	0.77~1.12	0.42	0.94	0.78~1.13	0.51	0.96	0.79~1.16	0.67	0.89	0.57~1.39	0.60	0.91	0.58~1.42	0.67	0.93	0.59~1.46	0.74
Pursuing one's interest	1.00	0.83~1.20	1.00	1.00	0.84~1.20	0.97	1.01	0.84~1.22	0.90	1.34	0.88~2.05	0.17	1.37	0.90~2.08	0.15	1.38	0.90~2.11	0.14
Social influence	0.93	0.78~1.11	0.43	0.93	0.77~1.11	0.40	0.93	0.78~1.11	0.43	0.75	0.50~1.15	0.19	0.77	0.50~1.16	0.21	0.77	0.50~1.19	0.24
Enduring active challenging	1.11	0.92~1.33	0.28	1.10	0.92~1.33	0.30	1.09	0.91~1.31	0.35	0.91	0.61~1.38	0.66	0.85	0.55~1.29	0.44	0.85	0.55~1.31	0.46
Cherishing family and friends	0.91	0.76~1.09	0.30	0.90	0.75~1.08	0.28	0.92	0.77~1.11	0.38	0.73	0.49~1.10	0.13	0.72	0.48~1.08	0.11	0.74	0.49~1.11	0.15
Graduating from school	0.93	0.80~1.08	0.34	0.93	0.80~1.09	0.38	0.96	0.82~1.12	0.56	0.67	0.47~0.96	0.03*	0.68	0.47~0.97	0.04*	0.69	0.48~1.00	0.05
Stable lifestyle	0.98	0.81~1.18	0.81	0.99	0.82~1.19	0.89	0.98	0.81~1.17	0.78	1.24	0.81~1.88	0.32	1.31	0.85~2.03	0.23	1.28	0.83~1.98	0.26
Commitment to values	1.05	1.00~1.10	0.07	1.05	1.00~1.10	0.08	1.04	0.99~1.09	0.15	0.94	0.84~1.06	0.32	0.94	0.83~1.06	0.30	0.94	0.83~1.06	0.28

§Odds Ratio (OR) for one-point increase of the score is shown

Sociodemographic variables were adjusted for in the Model 1; Smoke and Alcohol were additionally adjusted for in the Model 2; Living in 15 years old was additionally adjusted for in the Model 3

*p < 0.05, ** *p* < 0.01

According to Table 2, financial success was significantly and positively associated with suicidal ideation in the most recent year after adjusting for Model 1 and Model 2. However, there were no significant associations after adjusting for Model 3. As for Table 3, financial success was significantly and positively associated with suicidal planning over a lifetime after adjusting for Model 1 and Model 2. However, there were no significant associations after adjusting for Model 3. Financial success was also significantly and positively associated with suicidal planning in the most recent year after adjusting for Model 1. However, the significance disappeared after adjusting for Model 2. In Table 4, there were no significant associations between personal values in adolescence and suicide attempt over a lifetime and in the most recent year.

According to Table 2, graduating from school was significantly and negatively associated with suicidal ideation over a lifetime after adjusting for Model 1 and Model 2. Its significance disappeared after adjusting for Model 3. As for Table 3, graduating from school was significantly and negatively associated with suicidal ideation in the most recent year after adjusting for Model 1 and Model 2. Its significance disappeared after adjusting for Model 3. In Table 4, graduating from school was significantly and negatively associated with suicide attempt in the most recent year after adjusting for Model 1 and Model 2. Its significance disappeared after adjusting for Model 3.

Commitment to values was significantly and negatively associated with suicidal ideation in the most recent year after adjusting for socio-demographic variables. However, there were no significant associations with other suicidal outcomes.

## Discussion

To our knowledge, this is the first study to describe the association between personal values in adolescence and suicidality over a lifetime and in adulthood. Of the various priorities of values, cherishing family and friends was significantly and negatively associated with suicidal ideation over a lifetime and in the most recent year, and suicidal planning over a lifetime, even after the adjustment. Commitment to values was also significantly and negatively associated with suicidal ideation in the most recent year. The findings suggest that there is an association between personal value types and commitment and suicidal ideation, although the causality or direction of the association, if any, is still unknown.

The value priority on cherishing family and friends was significantly and negatively associated with suicidal ideation over a lifetime and in the most recent year, and suicide planning over a lifetime. The value priority on cherishing family and friends could be classified as a “benevolence” value area based on Schwartz’s theory of basic values [29]. Benevolence is defined as preserving and enhancing the welfare of those with whom one is in frequent personal contact, which is associated with behaviors such as help, honesty, not being resentful, loyalty, forgiving, friendship, and responsibility [29]. In a previous study, benevolence promoted cooperative and supportive social relations [29] and was positively associated with subjective well-being [25]. Our study’s findings are consistent with these findings. Having a value priority on cherishing family and friends during adolescence may prevent the development of suicidal ideation and planning at the least. However, our study findings were greatly limited by their retrospective nature. It is not clear if a value priority on cherishing family and friends during adolescence is useful in preventing suicide or attempted suicide. A further prospective study is needed to clarify the association between value priority on cherishing family and friends and suicidality.

The association between cherishing family and friends and suicidal ideation may further be explained by interpersonal theories of suicide, in which suicidal ideation is considered to occur when thwarted belongingness and perceived burdensomeness happen simultaneously [38]. Thwarted belongingness is the experience that one is alienated from others, not an integral part of a family, circle of friends, or other valued groups, and is characterized by loneliness and the absence of reciprocal care. Perceived burdensomeness is the view that one’s existence burdens family, friends, and/or society. Such a perception creates a sense of liability and self-hate. Those who put the most important value on cherishing family and friends could have a strong connection with their community, which deters loneliness, elicits reciprocal care, and likely decreases thwarted belongingness. In addition, those who have the value might have a lot of companions who will support them, which may lead them to experience success in society and may create high self-efficacy and self-esteem. These people are likely to feel they are less perceived as burdensome. Therefore, cherishing family and friends may work protectively against thwarted belongingness and perceived burdensomeness, which might weaken suicidal ideation.

Commitment to values was also significantly and negatively associated with suicidal ideation in the most recent year. A previous study revealed that commitment to values was associated with well-being [27, 28], a finding supported by this study. Our study also suggests that people who commit themselves to their own values may have higher self-efficacy and/or self-esteem, which would serve to mitigate the occurrence of suicidal ideation.

However, an alternative explanation should be considered: that suicidality may affect cognition or reporting of the personal values of respondents. A person who has considered suicide may also have negative cognitions of himself/herself, which may lead to a negative distortion of his/her recall of personal values during adolescence. In addition, a person who has had suicidal ideas may feel he/she did not place a high value on family and friends or did not have a high commitment to his/her values, *simply because he/she wished to die*. This feeling may lead him/her to perceive that he/she has placed a low value on cherishing family and friends or had less commitment to values since childhood. Moreover, the other explanation includes the possibility that suicidality and personal values in adolescence are influenced by a shared factor, such as past negative experiences. For instance, an experience of child abuse is known to increase the risk of suicidality [17] and may also decrease the value on cherishing family. Bullying at school may increase the risk of suicidality [39] and, at the same time, it may decrease the value on cherishing friends. Having a mental disorder in childhood can also raise the risk of suicidality in adulthood [40] and can also lower commitment to values. These alternative explanations/hypotheses need to be investigated in future research.

Cherishing family and friends was non-significantly associated with attempted suicide. This may be due to a small number of cases that reported attempted suicide. However, the OR was closer to 1 for suicide attempt than for other suicidalities. A previous study reported that most people who had suicidal ideation did not attempt suicide [6, 41]. While suicidal ideation, suicidal planning, and suicide attempt shared common risk factors [42], the risk factors could differ [6, 43, 44]. The finding that the value priority of cherishing family and friends is not associated with suicide attempt, was considered the reason for this gap.

The strengths of this study are as follows. First, a systematic sampling method was used, which could prevent selection bias. Second, this study dealt with personal values in adolescence, a subject that has not been fully researched, and has originality. Third, as a practical implication, the moral education of a cherishing family and friends during adolescence could serve to prevent suicide.

However, there were many limitations. First, the baseline response rate was low and there were some socio-demographic differences between those who responded to the wave 3 survey and those who did not: For instance, participants who were younger, male, not married, working, and with a high household income dropped out at wave 3. The association between personal values and suicidality may be underestimated if those subjects who had specific personal values and high suicidality had less participation in the surveys. Second, recall bias may have occurred because the participants had to remember an important value when they were 15 years old. In particular, there may be an information bias such as hindsight bias. Respondents who had experienced suicidality may have attributed their suicidality to their lack of value on cherishing family and friends or their commitment to values during adolescence, and reported accordingly. Third, childhood adversity could be a confounding factor because it affects suicidality and is assumed to be associated with personal values in adolescence. Fourth, the number of cases of suicide attempt as a proportion of our overall sample size was small. This may lead to a greater likelihood of type 2 error. Finally, since the validity and reliability of the measurement of personal values in adolescence used in this study has not been fully evaluated, random error may have been occurred.

## Conclusions

This retrospective study indicated a possible association between the personal value on cherishing family and friends and commitment to values during adolescence, on the one hand, and lifetime and one-year suicidal ideation, on the other hand. Investigating the association between personal values and suicidality may be a promising new approach to understanding and preventing suicide.

## Data Availability

The data that support the findings of this study are available from the corresponding author on request.

## Appendix

**Table 5:** Comparison of sociodemographic, health-related behaviors, and suicidality between respondents who completed the wave 1 and 3 surveys (*n* = 2787) and those who completed the wave 1 survey but did not the wave 3 survey (*n* = 1570)

	Response to wave 3		Attrition		t / χ^2^	*P* value
	N (%)	Mean (SD)	N (%)	Mean (SD)		
Age (mean)		38.08 (7.0)		36.0 (7.3)	9.28	< 0.01**
Sex (men)	1233 (44.2)		810 (50.7)		16.97	< 0.01**
Married (yes)	2087 (75.0)		955 (60.0)		107.86	< 0.01**
Education					8.76	0.03*
Junior high school	83 (3.0)		66 (4.2)			
High school	555 (20.2)		313 (20.0)			
Some college	958 (34.8)		491 (31.4)			
University or higher	1158 (42.0)		692 (44.3)			
Employment					10.66	< 0.01**
Working	2153 (77.6)		1283 (80.7)			
On leave	64 (2.3)		47 (3.0)			
Job seeking or House wife or Students	559 (20.1)		260 (16.4)			
Household Income (per year)					76.03	< 0.01**
≦2.5 million yen	194 (7.0)		113 (7.1)			
≦5 million yen	433 (15.5)		251 (15.7)			
≦7.5 million yen	636 (22.8)		272 (17.0)			
>7.5 million yen	882 (31.6)		413 (25.8)			
Not known	642 (23.0)		549 (34.4)			
Current or ever smoking (yes)	1258(45.2)		788 (49.4)		7.35	< 0.01**
Drinking alcohol					1.08	0.30
More than 3 times per week	938 (33.7)		514 (32.2)			
Less than 3 times per week	1842(66.3)		1082 (67.8)			
Economic status at age 15 (poor)	523 (18.9)		291 (18.2)		0.39	0.83
Suicidality						
Suicidal ideation over a lifetime (yes)	509 (18.4)		287 (18.3)		0.01	0.92
Suicidal ideation in the most recent year (yes)	144 (5.2)		84 (5.4)		0.05	0.83
Suicidal planning over a lifetime (yes)	159 (5.8)		98 (6.9)		0.46	0.50
Suicidal planning in the most recent year (yes)	45 (1.6)		34 (2.2)		1.65	0.20
Suicide attempt over a lifetime (yes)	116 (4.2)		88 (5.6)		4.50	0.03*
Suicide attempt in the most recent year (yes)	19 (0.7)		19 (1.2)		3.17	0.08

**p* < 0.05, ** *p* < 0.01

## Abbreviations

CAPI: Computer-aided personal instrument
J-SHINE: Japanese Study on Stratification, Health, Income, and Neighborhood
PVQ-57: 57-item Portrait Values Questionnaire
PVQ-II: Personal Values Questionnaire II
STROBE: Strengthening the Reporting of Observational Studies in Epidemiology
